# Hyper–Kähler Manifolds of Generalized Kummer Type and the Kuga–Satake Correspondence

**DOI:** 10.1007/s00032-022-00369-8

**Published:** 2022-10-19

**Authors:** M. Varesco, C. Voisin

**Affiliations:** 1 Universität Bonn: Rheinische Friedrich-Wilhelms-Universitat Bonn, Bonn, Germany; 2 Mathematics Institute of Jussieu-Paris Rive Gauche, Paris, France

## Abstract

We first describe the construction of the Kuga–Satake variety associated to a (polarized) weight-two Hodge structure of hyper-Kähler type. We describe the classical cases where the Kuga–Satake correspondence between a hyper-Kähler manifold and its Kuga–Satake variety has been proved to be algebraic. We then turn to recent work of O’Grady and Markman which we combine to prove that the Kuga–Satake correspondence is algebraic for projective hyper-Kähler manifolds of generalized Kummer deformation type.

## Introduction

The Kuga–Satake construction associates to any K3 surface, and more generally to any weight-two Hodge structure of hyper-Kähler type a complex torus which is an abelian variety when the Hodge structure is polarized. This construction allows to realize the Hodge structure on degree-two cohomology of a projective hyper-Kähler manifold as a direct summand of the $H^2$ of an abelian variety. Although the construction is formal and not known to be motivic, it has been used by Deligne in [2] to prove deep results of a motivic nature, for example the Weil conjecture for K3 surfaces can be deduced from the Weil conjectures for abelian varieties.

Section 2 of the notes is devoted to the description of the original construction and the presentation of a few classical examples where the Kuga–Satake correspondence is known to be algebraic, i.e., realized by a correspondence between the hyper-Kähler manifold and its Kuga–Satake variety. In Sect. 3, we focus on the case of hyper-Kähler manifolds of a generalized Kummer type and present a few recent results. If *X* is a (very general) projective hyper-Kähler manifold of generalized Kummer type, the Kuga–Satake variety $\mathrm{KS}(X)$ built on $H^2(X,{\mathbb {Z}})_{\mathrm{tr}}$ is a sum of copies of an abelian fourfold $\mathrm{KS}(X)_c$ of Weil type. There is another abelian fourfold associated to *X*, namely the intermediate Jacobian $J^3(X)$ which is defined as the complex torus

$$\begin{aligned} J^3(X)=H^{1,2}(X)/H^3(X,{\mathbb {Z}}) \end{aligned}$$

where $b_3(X)=8$. Here we use the fact that $H^{3,0}(X)=0$ and the projectivity of *X* guarantees that $J^3(X)$ is an abelian variety. O’Grady [11] proves the following result.

### Theorem 1.1

The two abelian varieties $$J^3(X) $$ and $$\mathrm{KS}(X)_c$$ are isogenous.

We also prove in Sect. 3.2 a more general statement concerning hyper-Kähler manifolds with $b_3(X)\not =0$. Section 3.3 is devoted to the question of the algebraicity of the Kuga–Satake correspondence. Following [20], we prove, using a theorem of Markman and Theorem 1.1 above that the Kuga–Satake correspondence is algebraic for hyper-Kähler manifolds of generalized Kummer type.

### Theorem 1.2

There exists a codimension-2*n* cycle ${\mathcal {Z}}\in \mathrm{CH}^{2n}(\mathrm{KS}(X)_c\times X)_{{\mathbb {Q}}}$ such that

1.1 $$\begin{aligned}{}[{\mathcal {Z}}]_*: H_2(\mathrm{KS}(X)_c,{\mathbb {Q}})\rightarrow H_2(X,{\mathbb {Q}})\end{aligned}$$

is surjective.

## The Kuga–Satake Construction

### Main Construction

In this section, we recall the construction and some properties of the Kuga–Satake variety associated to a Hodge structure of *hyper-Kähler type*. This construction is due to Kuga and Satake in [6]. For a complete introduction see [15] and [4, Ch. 4].

#### Definition 2.1

A pair (*V*, *q*) is a Hodge structure of *hyper-Kähler type* if the following conditions hold: *V* is a rational level-two Hodge structure with $\dim V^{2,0}=1$, and $q:V\otimes V\rightarrow {\mathbb {Q}}(-2)$ is a morphism of Hodge structures whose real extension is negative definite on $(V^{2,0}\oplus V^{0,2})\cap V_{\mathbb {R}}$.

#### Remark 2.2

Note that if *X* is a hyper-Käler manifold and $q_X$ is the Beauville-Bogomolov quadratic form, the pair $(H^2(X,{\mathbb {Q}}),-q_X)$ is indeed a Hodge structure of *hyper-Kähler type*.

Let (*V*, *q*) be a Hodge structure of hyper-Kähler type, and let *T*(*V*) be the tensor algebra of the underlying rational vector space *V*:

$$\begin{aligned} T(V):=\bigoplus _{i\ge 0}V^{\otimes i} \end{aligned}$$

where $V^{\otimes 0}:={\mathbb {Q}}$. The *Clifford algebra* of (*V*, *q*) is the quotient algebra

$$\begin{aligned} \mathrm {Cl}(V):=\mathrm {Cl}(V,q):=T(V)/I(q),\end{aligned}$$

where *I*(*q*) is the two-sided ideal of *T*(*V*) generated by elements of the form $v\otimes v-q(v)$ for $v\in V$. Since *I*(*q*) is generated by elements of even degree, the natural ${\mathbb {Z}}/2{\mathbb {Z}}$-grading on *T*(*V*) induces a ${\mathbb {Z}}/2{\mathbb {Z}}$-grading on $\mathrm {Cl}(V)$. Write

$$\begin{aligned}\mathrm {Cl}(V)=\mathrm {Cl}^+(V)\oplus \mathrm {Cl}^-(V),\end{aligned}$$

where $\mathrm {Cl}^+(V)$ is the even part and $\mathrm {Cl}^-(V)$ is the odd part. Note that $\mathrm {Cl}^+(V)$ is still a ${\mathbb {Q}}$-algebra, it is called *the even Clifford algebra*.

We now use the assumption that (*V*, *q*) is a Hodge structure of hyper-Kähler type to define a complex structure on $\mathrm {Cl}^+(V)_{\mathbb {R}}$. Consider the decomposition of the real vector space $V_{\mathbb {R}}=V_1\oplus V_2,$ with

$$\begin{aligned} \quad V_1:=V^{1,1}\cap V_{\mathbb {R}},\quad V_2:=\{V^{2,0}\oplus V^{0,2}\}\cap V_{\mathbb {R}}. \end{aligned}$$

The ${\mathbb {C}}$-linear span of $V_2$ is the two-dimensonal vector space $V^{2,0}\oplus V^{0,2}$. Therefore, *q* is negative definite on $V_2$. Pick a generator $\sigma =e_1+ie_2$ of $V^{2,0}$ with $e_1, e_2\in V_2$ and $q(e_1)=-1$. Since $q(\sigma )=0$, we deduce that $q(e_1,e_2)=0$ and $q(e_2)=-1$, i.e., $\{e_1,e_2\}$ is an orthonormal basis of $V_2$. From this, it is straightforward to check that $e_1\cdot e_2=-e_2\cdot e_1$ in $\mathrm {Cl}(V)_{\mathbb {R}}$. Therefore, $J:=e_1\cdot e_2$ satisfies the equation $J^2=-1$ and left multiplication by *J* induces a complex structure on the real vector space $\mathrm {Cl}(V)_{\mathbb {R}}$ which preserves the real subspaces $\mathrm {Cl}^+(V)_{\mathbb {R}}$ and $\mathrm {Cl}^-(V)_{\mathbb {R}}$. Giving a complex structure on a real vector space is equivalent to giving a Hodge structure of level one on the rational vector space:

#### Definition 2.3

The *Kuga–Satake Hodge structure* $H^1_{\mathrm{KS}}(V)$ is the Hodge structure of level one on $\mathrm {Cl}^+(V)$ given by

$$\begin{aligned} \rho :{\mathbb {C}}^*\rightarrow \mathrm {GL}(\mathrm {Cl}^+(V)_{\mathbb {R}}),\quad x+yi\rightarrow x+yJ, \end{aligned}$$

where $x+yJ$ acts on $\mathrm {Cl}^+(V)_{\mathbb {R}}$ via left multiplication.

Therefore, starting from a rational level-two Hodge structure of hyper-Kähler type (*V*, *q*), we constructed a rational Hodge structure of level one on $\mathrm {Cl}^+(V)$. This determines naturally a complex torus up to isogeny: let $\Gamma \subseteq \mathrm {Cl}^+(V)$ be a lattice in the rational vector space $\mathrm {Cl}^+(V)$. Then, the *Kuga–Satake variety* associated to (*V*, *q*) is the (isogeny class of) the complex torus

$$\begin{aligned} \mathrm {KS}(X):=\mathrm {Cl}^+(V)_{\mathbb {R}}/\Gamma , \end{aligned}$$

where $\mathrm {Cl}^+(V)_{\mathbb {R}}$ is endowed with the complex structure induced by left multiplication by *J*. Note that if (*V*, *q*) is an integral Hodge structure of hyper-Kähler type, then *V* can be viewed as a lattice in $\mathrm {Cl}^+(V_{\mathbb {Q}})$. Thus, the natural choice $\Gamma :=V$ determines the complex torus $\mathrm {KS}(V)$, and not just up to isogeny.

By construction, one has the following:

$$\begin{aligned} H^1_\mathrm {KS}(V):=H^1(\mathrm {KS}(V),{\mathbb {Q}}) \simeq \mathrm {Cl}^+(V)^*\simeq \mathrm {Cl}^+(V), \end{aligned}$$

where the isomorphism between $\mathrm {Cl}^+(V)$ and its dual is induced by the nondegenerate form *q*.

#### Remark 2.4

Consider the case where *V* can be written as a direct sum of Hodge structures $V=V_1\oplus V_2$. Since $\dim V^{2,0}=1$, either $V_1$ or $V_2$ has to be pure of type (1, 1). We may then assume that $V_2^{2,0}=0$. In this case, one checks that the Kuga–Satake Hodge structure $\mathrm {Cl}^+(V)$ is isomorphic to the product of $2^{n_2-1}$ copies of $\mathrm {Cl}^+(V_1)\oplus \mathrm {Cl}^-(V_1)$, with $n_2:=\dim V_2$. In particular:

$$\begin{aligned} \mathrm {KS}(V_1\oplus V_2)\sim \mathrm {KS}(V_1)^{2^{n_2}}. \end{aligned}$$

#### Remark 2.5

For any element $w\in \mathrm {Cl}^+(V)$, the right-multiplication morphism

$$\begin{aligned} r_w:\mathrm {Cl}^+(V)\rightarrow \mathrm {Cl}^+(V), \quad r_w(x):=x\cdot w \end{aligned}$$

is a morphism of Hodge structures. This follows from the fact that the Kuga–Satake Hodge structure on $\mathrm {Cl}^+(V)$ is induced by left multiplication by $J\in \mathrm {Cl}^+(V)$ which commutes with right multiplication by elements of $\mathrm {Cl}^+(V)$. Therefore, there is an embedding

$$\begin{aligned} \mathrm {Cl}^+(V)\hookrightarrow \mathrm {End}_\mathrm {Hdg}(\mathrm {Cl}^+(V))\simeq \mathrm {End}(\mathrm {KS}(V))\otimes {\mathbb {Q}}. \end{aligned}$$

Since the dimension of $\mathrm {Cl}^+(V)$ is $2^{\dim V-1}$, we deduce that the endomorphism algebra of $\mathrm {KS}(V)$ is in general big. This is related with the fact that the Kuga–Satake variety of a Hodge structure of hyper-Kähler type is in general not simple, but it is isogenous to the power of a smaller-dimensional torus.

Remarkably, the Kuga–Satake construction realizes the starting level-two Hodge structure as a Hodge substructure of the tensor product of two Hodge structures of level one:

#### Theorem 2.6

Let (*V*, *q*) be a Hodge structure of hyper-Kähler type. Then, there is an embedding of Hodge structures:

$$\begin{aligned} V\hookrightarrow \mathrm {Cl}^+(V)\otimes \mathrm {Cl}^+(V), \end{aligned}$$

where $\mathrm {Cl}^+(V)$ is endowed with the level-one Hodge structure of Definition 2.3.

#### Proof

We recall here just the definition of the desired map, for more details we refer to [4, Prop. 3.2.6]. Fix an element $v_0\in V$ such that $q(v_0)\not =0$ and consider the following left multiplication map:

$$\begin{aligned} \varphi :V\rightarrow \mathrm {End}(\mathrm {Cl}^+(V)),\quad v\mapsto f_v, \end{aligned}$$

where $f_v(w):=v\cdot w\cdot v_0$. The injectivity of $\varphi$ follows from the equality $f_v(v'\cdot v_0)=q(v_0)(v\cdot v')$ for any $v'\in V$. See the reference above for the proof of the fact that $\varphi$ is a morphism of Hodge structures. Finally, the desired embedding is given by the composition of $\phi$ and the isomorphisms

$$\begin{aligned} \mathrm {End}(\mathrm {Cl}^+(V))\simeq \mathrm {Cl}^+(V)^*\otimes \mathrm {Cl}^+(V)\simeq \mathrm {Cl}^+(V)\otimes \mathrm {Cl}^+(V), \end{aligned}$$

where the isomorphism $\mathrm {Cl}^+(V)^*\simeq \mathrm {Cl}^+(V)$ is induced by *q*. $\square$

#### Remark 2.7

Note that the embedding of Theorem 2.6 depends on the choice of $v_0\in V$. Nevertheless, choosing another $v_0'\in V$ changes the embedding by the automorphism of $\mathrm {Cl}^+(V)$ which sends *w* to $\frac{ w\cdot v_0\cdot v_0'}{q(v_0)}$.

Theorem 2.6 shows that any Hodge structure of hyper-Kähler type can be realized as a Hodge substructure of $W\otimes W$ for some level-one Hodge structure *W*. On the other hand, in [2, Sec. 7], Deligne proves that the same conclusion does not hold for a very general level-two Hodge structure. We recall here a version of this fact as presented in [15, Prop. 4.2].

#### Theorem 2.8

Let (*V*, *q*) be a polarized level-two Hodge structure whose Mumford–Tate group $\mathrm {MT}(V)$ is maximal, that is, equal to $\mathrm {SO}(q)$. If $\dim V^{2,0}>1$, then *V* cannot be realized as a Hodge substructure of $W\otimes W$ for any level-one Hodge structure *W*.

#### Remark 2.9

One can show in some cases that the technical condition $\mathrm {MT}(V)=\mathrm {SO}(q)$ of Theorem 2.8 is satisfied for a very general Hodge structure, see [2, Sec. 7] and [19, Cor. 4.12]. The proof goes as follows: Given a smooth projective morphism $\pi :{\mathcal {X}}\rightarrow B$, one shows that for very general $t\in B$, the Mumford–Tate group $\mathrm {MT}({\mathcal {X}}_t)$ contains a finite index subgroup of the monodromy group of the base. Already in the case of hypersurfaces in a $(2r+1)$-dimensional projective space, this shows that for a very general hypersurface $X_s$, the Mumford–Tate group of $H^{2r}(X,{\mathbb {Q}})$ is maximal in the above sense. Applying Theorem 2.8, one then sees that the second cohomology of a very general surface *X* in ${\mathbb {P}}^3$ of degree $\ge 5$ cannot be realized as a Hodge substructure of $W\otimes W$ for any level-one Hodge structure *W*.

To conclude this section, we recall the fact that if the Hodge structure of hyper-Kähler type is polarized, the resulting Kuga–Satake Hodge structure on the even Clifford algebra is naturally polarized.

#### Theorem 2.10

If (*V*, *q*) is a Hodge structure of hyper-Kähler type such that *q* is a polarization for *V*, then the Kuga–Satake Hodge structure on $\mathrm {Cl}^+(V)$ has a natural polarization. In particular, the Kuga–Satake torus $\mathrm {KS}(V)$ is an abelian variety.

### Some Examples

Let *X* be a hyper-Kähler variety (resp. a two-dimensional complex torus). The pair $(H^2(X,{\mathbb {Q}}),-q_X)$ where $q_X$ is the Beauville–Bogomolov form (resp. the intersection pairing) is a Hodge structure of hyper-Kähler type. Therefore, we can apply the Kuga–Satake construction to it and we get the *Kuga–Satake variety* of *X*:

$$\begin{aligned} \mathrm {KS}(X):=\mathrm {KS}(H^2(X,{\mathbb {Q}})). \end{aligned}$$

Since $-q_X$ is not a polarization on the whole $H^2(X,{\mathbb {Q}})$, the variety $\mathrm {KS}(X)$ is not necessarily an abelian variety, but it is just a complex torus. On the other hand, if *X* is projective and *l* is an ample class on *X*, the primitive part

$$\begin{aligned} H^2(X,{\mathbb {Q}})_p:=l^{\bot }\subseteq H^2(X,{\mathbb {Q}}) \end{aligned}$$

is a Hodge substructure which is polarized by the restriction of the form $-q_X$. Therefore, by Theorem 2.10, the Kuga–Satake variety of $H^2(X,{\mathbb {Q}})_p$ is an abelian variety. Moreover, by Remark 2.4, we have

$$\begin{aligned} \mathrm {KS}(X):=\mathrm {KS}(H^2(X,{\mathbb {Q}}))\sim \mathrm {KS}(H^2(X,{\mathbb {Q}})_p)^2. \end{aligned}$$

In particular, in the projective case, $\mathrm {KS}(X)$ is an abelian variety. A similar argument can be applied to $H^2(X,{\mathbb {Q}})_\mathrm {tr}\subseteq H^2(X,{\mathbb {Q}})$, the transcendental lattice of a projective K3 surface, to deduce that $\mathrm {KS}(X)$ is isogenous to some power of the abelian variety $\mathrm {KS}(H^2(X,{\mathbb {Q}})_\mathrm {tr})$. On the other hand, if *X* is not projective, the torus $\mathrm {KS}(X)$ need not be polarized.

#### Theorem 2.11

[10] Let *A* a complex torus of dimension two. Then, there exists an isogeny

$$\begin{aligned} \mathrm {KS}(A)\sim (A\times {\hat{A}})^4, \end{aligned}$$

where ${\hat{A}}$ is the dual complex torus. In particular, if *A* is an abelian surface

$$\begin{aligned} \mathrm {KS}(A)\sim A^8\quad \text {and} \quad \mathrm {KS}(\mathrm {Kum}(A))\sim A^{2^{19}},\end{aligned}$$

where $\mathrm {Kum}(A)$ is the Kummer surface associated to *A*.

#### Definition 2.12

Let *A* be an abelian variety of dimension 2*n* and let *d* be a positive real number. Then, *A* is called of ${\mathbb {Q}}(\sqrt{-d})$*-Weil type* if ${\mathbb {Q}}(\sqrt{-d})\subseteq \mathrm {End}(A)\otimes _{\mathbb {Z}}{\mathbb {Q}}$ and if the action of $\sqrt{-d}$ on the tangent space at the origin of *A* has eigenvalues $\sqrt{-d}$ and $-\sqrt{-d}$ both with multiplicity *n*.

Given an abelian of ${\mathbb {Q}}(\sqrt{-d})$-Weil type *A*, then one can associate naturally an element $\delta \in {\mathbb {Q}}/\mathrm {N}({\mathbb {Q}}(\sqrt{-d}))$, where $\mathrm {N}({\mathbb {Q}}(\sqrt{-d}))$ is the set of norms of ${\mathbb {Q}}(\sqrt{-d})$. The element $\delta$ is called the *discriminant* of *A*. Abelian varieties of Weil type appear often as simple factors of Kuga–Satake varieties; the next result due to Lombardo [7] gives an example of this fact. We recall here the version presented in [15, Thm. 9.2]. In the following, *U* denotes the hyperbolic plane.

#### Theorem 2.13

Let *d* be a positive real number and let *A* be an abelian fourfold of ${\mathbb {Q}}(\sqrt{-d})$-Weil type of discriminant $\delta =1$. Then, $A^4$ is the Kuga–Satake variety of a polarized Hodge structure of hyper-Kähler type of dimension six (*V*, *q*), such that

$$\begin{aligned} V\simeq U^{\oplus 2}\oplus \langle -1 \rangle \oplus \langle -d \rangle \quad \end{aligned}$$

as quadratic spaces. Conversely, if (*V*, *q*) is a Hodge structure of hyper-Kähler type of dimension six as above, its Kuga–Satake variety is isogenous to $A^4$ for some abelian fourfold of ${\mathbb {Q}}(\sqrt{-d})$-Weil type.

### Kuga–Satake Hodge Conjecture

In this section, we analyze the morphism of Hodge structures

$$\begin{aligned} V\hookrightarrow \mathrm {Cl}^+(V)\otimes \mathrm {Cl}^+(V) \end{aligned}$$

of Theorem 2.6, in the case where $V=H^2(X,{\mathbb {Q}})_\mathrm {tr}$, the transcendental lattice of a projective hyper-Kähler variety *X*. Using the isomorphism $\mathrm {Cl}^+(H^2(X,{\mathbb {Q}})_\mathrm {tr})\simeq H^1_\mathrm {KS}(H^2(X,{\mathbb {Q}})_\mathrm {tr})$, we apply the Künneth decomposition and obtain an embedding

$$\begin{aligned} H^1_\mathrm {KS}(H^2(X,{\mathbb {Q}})_\mathrm {tr})\otimes H^1_\mathrm {KS}(H^2(X,{\mathbb {Q}})_\mathrm {tr})\hookrightarrow H^2(\mathrm {KS}(H^2(X,{\mathbb {Q}})_\mathrm {tr})^2,{\mathbb {Q}}). \end{aligned}$$

On the other hand, since we the variety *X* is projective there is a natural projection map $H^2(X,{\mathbb {Q}})\rightarrow H^2(X,{\mathbb {Q}})_\mathrm {tr}$. Composing these morphisms, we obtain the morphism of Hodge structures

$$\begin{aligned} H^2(X,{\mathbb {Q}})\rightarrow H^2(\mathrm {KS}(H^2(X,{\mathbb {Q}})_\mathrm {tr})^2,{\mathbb {Q}}), \end{aligned}$$

which is called the *Kuga–Satake correspondence*. This morphism corresponds via Poincaré duality to a Hodge class

$$\begin{aligned} \kappa \in H^{2n,2n}(X\times \mathrm {KS}(H^2(X,{\mathbb {Q}})_\mathrm {tr})\times \mathrm {KS}(H^2(X,{\mathbb {Q}})_\mathrm {tr})), \end{aligned}$$

where $2n=\dim X$. The Hodge conjecture applied to this special case gives us the following:

#### Conjecture 2.14

(Kuga–Satake Hodge conjecture) Let *X* be a projective hyper-Kähler variety or a complex projective surface with $h^{2,0}=1$. Then, the class $\kappa$ is algebraic.

#### Remark 2.15

In the case where *X* is an abelian surface or a Kummer surface, the Kuga–Satake Hodge conjecture can be deduced from Theorem 2.11, using the fact that the Hodge conjecture is known for self-products of any given abelian surface [9].

The Kuga–Satake Hodge conjecture is not known in most cases, already in the case of K3 surfaces. One of the very few examples for which it has been proved is the family of K3 surfaces studied by Paranjape in [12]: let $L_1,\ldots , L_6$ be six lines in ${\mathbb {P}}^2$ no three of which intersect in one point, and let $\pi :Y\rightarrow {\mathbb {P}}^2$ be the double cover of ${\mathbb {P}}^2$ branched along the six lines. Then, *Y* is a singular surface with simple nodes in the preimages of the intersection points of the lines $L_i$. Resolving the singularities of $\pi$ by blowing up the nodes one obtains a K3 surface *X*. For a general choice of the six lines, the Picard number of *X* is equal to 16, where a basis of the Néron–Severi group is given by the 15 exceptional divisors over the singular points of *Y*, together with the pullback of the ample line of ${\mathbb {P}}^2$ via the map $X\rightarrow {\mathbb {P}}^2$. In particular, the transcendental lattice of *X* is six-dimensional, and satisfies the hypotheses of Theorem 2.13. Its Kuga–Satake variety is therefore isogenous to the fourth power of some abelian fourfold. In [12], the author shows that this abelian fourfold is the Prym variety of some 4 : 1 cover $C\rightarrow E$ where *C* is a genus 5 curve and *E* is an elliptic curve, and finds a cycle in the product of *X* and the Prym variety which realizes the Kuga–Satake correspondence.

The fact that the Kuga–Satake correspondence is algebraic for the family described above has been used by Schlickewei to prove the Hodge conjecture for the square of those K3 surfaces:

#### Theorem 2.16

[14, Thm. 2] Let *X* be a K3 surface which is the desingularization of a double cover of ${\mathbb {P}}^2$ branched along six lines no three of which intersect in one point. Then, the Hodge conjecture is true for $X^2$.

In [5], the Kuga–Satake Hodge conjecture is proved for K3 surfaces which are desingularization of singular K3 surfaces in ${\mathbb {P}}^4$ with 15 nodal points. The authors then show that the same techniques as in Theorem 2.16 can be used to prove the Hodge conjecture for the square of these K3 surfaces.

#### Theorem 2.17

[5] Let *X* be a K3 surface which is the desingularization of a singular K3 surface in ${\mathbb {P}}^4$ with 15 nodal points. Then, the Kuga–Satake Hodge conjecture holds for *X* and the Hodge conjecture is true for $X^2$.

As a part of its PhD thesis, the first author of these notes generalize these two results and proves the following:

#### Theorem 2.18

[17, Thm. 4.3] Let ${\mathscr {X}}\rightarrow S$ be a four-dimensional family of K3 surfaces whose general fibre is of Picard number 16 with an isometry

$$\begin{aligned} T({\mathscr {X}}_s)\simeq U^2\oplus \langle a\rangle \oplus \langle b\rangle , \end{aligned}$$

for some negative integers *a* and *b*. If the Kuga–Satake correspondence is algebraic for the fibres of this family, then the Hodge conjecture holds for all powers of every K3 surface in this family.

The families of K3 surfaces studied in [14] and in [5] satisfy the hypotheses of Theorem 2.18. Therefore, Theorem 2.18 shows in particular that the Hodge conjecture holds for all powers of the K3 surfaces in the two families considered in [5, 14]. The techniques applied are similar to the one introduced in [14] with the addition of a deformation argument which allows to prove the Hodge conjecture for all powers of the K3 surfaces of higher Picard number in the family.

In the next section, we review another type of polarized hyper-Kähler manifolds for which the Kuga–Satake Hodge conjecture can be proved: The family of hyper-Kähler manifolds of generalized Kummer type.

## The Case of Hyper-Kähler Manifolds of Generalized Kummer Type

### Cup-Product: Generalization of a Result of O’Grady

Let *X* be a hyper-Kähler manifold of dimension 2*n* with $n\ge 2$. The Beauville–Bogomolov quadratic form $q_X$ is a nondegenerate quadratic form on $H^2(X,{\mathbb {Q}})$, whose inverse gives an element of $\mathrm{Sym}^2H^2(X,{\mathbb {Q}})$. By Verbitsky [18], the later space imbeds by cup-product in $H^4(X,{\mathbb {Q}})$, hence we get a class

3.1 $$\begin{aligned} c_X\in H^4(X,{\mathbb {Q}}). \end{aligned}$$

The O’Grady map $\phi :\bigwedge ^2H^3(X,{\mathbb {Q}})\rightarrow H^{4n-2}(X,{\mathbb {Q}})$ is defined by

3.2 $$\begin{aligned} \phi (\alpha \wedge \beta )=c_X^{n-2}\cup \alpha \cup \beta . \end{aligned}$$

The following result was first proved by O’Grady [11] in the case of a hyper-Kähler manifold of generalized Kummer deformation type.

#### Theorem 3.1

[11, 20] Let *X* be a hyper-Kähler manifold of dimension 2*n*. Assume that $H^3(X,{\mathbb {Q}})\not =0$. Then, the O’Grady map map $\phi :\bigwedge ^2H^3(X,{\mathbb {Q}})\rightarrow H^{4n-2}(X,{\mathbb {Q}})$ is surjective.

#### Proof

We can choose the complex structure on *X* to be general, so that $\rho (X)=0$. This implies that the Hodge structure on $H^2(X,{\mathbb {Q}})$ (or equivalently $H^{4n-2}(X,{\mathbb {Q}})$ as they are isomorphic by combining Poincaré duality and the Beauville-Bogomolov form) is simple. As the morphism $\phi$ is a morphism of Hodge structures, its image is a Hodge substructure of $H^{4n-2}(X,{\mathbb {Q}})$, hence either $\phi$ is surjective, or it is 0. Theorem 3.1 thus follows from the next proposition.$\square$

#### Proposition 3.2

The map $\phi$ is not identically 0.

#### Sketch of proof

Let $\omega \in H^2(X,{\mathbb {R}})$ be a Kähler class. Then, we know that the $\omega$-Lefschetz intersection pairing $\langle \,,\,\rangle _\omega$ on $H^3(X,{\mathbb {R}})$, defined by

$$\begin{aligned} \langle \alpha ,\beta \rangle _\omega :=\int _X\omega ^{2n-3}\cup \alpha \cup \beta \end{aligned}$$

is nondegenerate. This implies that the image of the map

$$\begin{aligned} \psi :\bigwedge ^2H^3(X,{\mathbb {Q}})\rightarrow H^6(X,{\mathbb {Q}}) \end{aligned}$$

pairs nontrivially with the image of $\mathrm{Sym}^{2n-3}H^2(X,{\mathbb {Q}})$ in $H^{4n-6}(X,{\mathbb {Q}})$. Note that the Hodge structure on $H^3(X,{\mathbb {Q}})$ has Hodge level one, so that the Hodge structure on the image of $\mathrm{Im}\,\psi$ in $\mathrm{Sym}^{2n-3}H^2(X,{\mathbb {Q}})^*$ is a Hodge structure of level at most two. One checks by a Mumford–Tate group argument (see [20] for more details) that, for a very general complex structure on *X*, the only level-two Hodge substructure of $\mathrm{Sym}^{2n-3}H^2(X,{\mathbb {Q}})$ is $c_X^{n-2} H^2(X,{\mathbb {Q}})$, where we see here $c_X$ as an element of $\mathrm{Sym}^2H^2(X,{\mathbb {Q}})$. It follows that the image of $\mathrm{Im}\,\psi$ in $\mathrm{Sym}^{2n-3}H^2(X,{\mathbb {Q}})^*$ pairs nontrivially with $c_X^{n-2} H^2(X,{\mathbb {Q}})$, which concludes the proof. $\square$

### Intermediate Jacobian and the Kuga–Satake Variety

#### Universal Property of the Kuga–Satake Construction

The following result is proved in [1]. Using the Mumford–Tate group, this is a statement in representation theory of the orthogonal group.

##### Theorem 3.3

Let $(H^2,q)$ be a polarized Hodge structure of hyper-Kähler type. Assume that the Mumford–Tate group of the Hodge structure on $H^2$ is maximal (that is, equal to the orthogonal group of *q*). Let *H* be a simple effective weight-one Hodge structure, such that there exists an injective morphism of Hodge structures of bidegree $(-1,-1)$

$$\begin{aligned} H^2\hookrightarrow \mathrm{End}(H). \end{aligned}$$

Then, *H* is a direct summand of the Kuga–Satake Hodge structure $H^1_{\mathrm{KS}}(H^2,q)$.

##### Idea of the proof

Let $G:=\mathrm {MT}(\mathrm {End}(H))$ and denote by ${\mathfrak {g}}$ its Lie algebra. Note that the group *G* acts on $H^2$, since $H^2$ is a Hodge substructure of $\mathrm {End}(H)$. Using the fact that the action of *G* preserves the polarization on $H^2$ and the hypothesis $\mathrm {MT}(H^2)=\mathrm {SO}(H^2)$, one sees that the image of *G* in $\mathrm {GL}(H^2)$ is $\mathrm {SO}(H^2)$. As $\mathfrak {so}(H^2)$ is a simple Lie algebra, we conclude that there exists a simple factor ${\mathfrak {g}}_0$ of the Lie algebra ${\mathfrak {g}}$ that maps isomorphically onto $\mathfrak {so}(H^2)$. Note that *G* is naturally a subgroup of $\mathrm {MT}(H)$, which is contained in $\mathrm {CSp(H)}$, the group generated by the symplectic group and the homotheties of *H*. In particular, there is a morphism of Lie algebras:

3.3 $$\begin{aligned} \mathfrak {so}(H^2)\simeq {\mathfrak {g}}_0 \hookrightarrow \mathfrak {sp}(H). \end{aligned}$$

By the classification result presented in [13] and explained in [3, 1.3.5–1.3.9], one concludes that the only embeddings as in (3.3) which correspond to irreducible representations of $\mathrm {SO}(V)$ are the spin representations. This proves that *H* is a direct summand of $H^1_{\mathrm{KS}}(H^2,q)$. $\square$

Charles’ theorem is in fact stronger, as it proves a similar statement for all tensor powers $H^{\otimes k}\otimes (H^*)^{\otimes k+2r}$. It also addresses the nonpolarized case that appears when dealing with nonprojective hyper-Kähler manifolds. In [16], another version of the universality property is proved. Namely

##### Theorem 3.4

Let $(H^2,q)$ be a polarized Hodge structure of hyper-Kähler type. Assume that $\dim H^2\ge 5$ and that the Mumford–Tate group of the Hodge structure on $H^2$ is maximal. Let *H* be a simple effective weight-one Hodge structure, such that there exists an injective morphism of Hodge structures of bidegree $(-1,-1)$

$$\begin{aligned} H^2\hookrightarrow \mathrm{Hom}(H,A), \end{aligned}$$

for some weight-one Hodge structure *A*. Then, *H* is a direct summand of the Kuga–Satake Hodge structure $H^1_{\mathrm{KS}}(H^2,q)$.

Coming back to Theorem 3.3, under the same assumption on the Mumford–Tate group, one knows that the Kuga–Satake weight-one Hodge structure is a power of a simple weight-one Hodge structure of dimension $\ge 2^{\lfloor \frac{b_2-1}{2}\rfloor }$, where $b_2=\mathrm{dim}\,H^2$, hence one gets as a consequence an inequality (see [1] for a more precise estimate)

$$\begin{aligned} \mathrm{dim}\,H\ge 2^{\lfloor \frac{b_2-1}{2}\rfloor }. \end{aligned}$$

##### Proof of Theorem 1.1

Let *X* be a very general projective hyper-Kähler manifold of generalized Kummer type of dimension $\ge 4$. We apply Theorem 3.3 to the O’Grady map (3.2) that we know to be a surjective morphism of Hodge structures by Theorem 3.1, or rather to its dual. We then conclude that $H^3(X,{\mathbb {Q}})$ contains a direct summand of $H^1_{\mathrm{KS}}(H^2(X,{\mathbb {Q}})_{\mathrm{tr}})$. As $H^1_{\mathrm{KS}}(H^2(X,{\mathbb {Q}})_{\mathrm{tr}})$ is a power of a simple weight-one Hodge structure $H^1_{\mathrm{KS}}(H^2(X,{\mathbb {Q}})_{\mathrm{tr}})_c$ of dimension 8, and $b_3(X)=8$, we conclude that $H^3(X,{\mathbb {Q}})\cong H^1_{\mathrm{KS}}(H^2(X,{\mathbb {Q}})_{\mathrm{tr}})_c$ as rational Hodge structures. $\square$

### Algebraicity of the Kuga–Satake Correspondence for Hyper-Kähler Manifolds of Generalized Kummer Type

#### Markman’s Result

For a projective manifold *X* with $h^{3,0}(X)\not =0$, it is expected from the Hodge conjecture that there exists a cycle ${\mathcal {Z}}\in \mathrm{CH}^2(J^3(X)\times X)_{\mathbb {Q}}$ such that $[{\mathcal {Z}}]_*:$ $H_1(J^3(X),{\mathbb {Q}})\rightarrow H^3(X,{\mathbb {Q}})$ is the natural isomorphism. Indeed, the map $[{\mathcal {Z}}]_*$ is an isomorphism of Hodge structures, hence provides a degree-4 Hodge class on $J^3(X)\times X$. Equivalently, after replacing ${\mathcal {Z}}$ by a multiple that makes it integral, the Abel–Jacobi map

$$\begin{aligned} \Phi _{\mathcal {Z}}:J^3(X)\rightarrow J^3(X),\quad \Phi _{\mathcal {Z}}:=\Phi _X\circ {\mathcal {Z}}_*,\end{aligned}$$

is a multiple of the identity and in particular $\Phi _X$ is surjective.

##### Theorem 3.5

(Markman [8]) Let *X* be a projective hyper-Kähler manifold of generalized Kummer type. Then, there exists a codimension-two cycle ${\mathcal {Z}}\in \mathrm{CH}^2(J^3(X)\times X)_{\mathbb {Q}}$ satisfying the property above.

The proof of this theorem uses a deformation argument starting from a generalized Kummer manifold, using the fact that $J^3(X)$ can be realized as a moduli space of sheaves on *X* in that case.

We now use Markman’s result to prove Theorem 1.2.

##### Proof of Theorem 1.2

Let ${\mathcal {Z}}$ be the Markman codimension-two cycle of Theorem 3.5. We choose a cycle $C_X\in \mathrm{CH}^2(X)_{\mathbb {Q}}$ of class $[C_X]=c_X$ (it exists by results of Markman [8]). We now consider the cycle

$$\begin{aligned} \Gamma ={\mathcal {Z}}^2\cdot \mathrm{pr}_X^*C_X^{n-2}\in \mathrm{CH}^{2n}(J^3(X)\times X){\mathbb {Q}}.\end{aligned}$$

One checks using the Künneth decomposition (see [20] for more details) that

$$\begin{aligned}{}[\Gamma ]_*:H_2(J^3(X),{\mathbb {Q}})\rightarrow H_2(X,{\mathbb {Q}}) \end{aligned}$$

is the O’Grady map. By Theorem 1.1, this is also the surjective morphism of Hodge structures (1.1). $\square$
